# Out of Academics: Education, Entrepreneurship and Enterprise

**DOI:** 10.1007/s10439-013-0839-x

**Published:** 2013-06-25

**Authors:** Albert J. Banes

**Affiliations:** 1 Joint Department of Biomedical Engineering, Biomedical Engineer ing Department, The University of North Carolina at Chapel Hill and North Carolina State University, Chapel Hill, NC 27514 USA; 2 Flexcell International Corp., 437 Dimmock’s Mill Rd., Hillsborough, NC 27278 USA

**Keywords:** Engineers, Entrepreneurs, Mechanobiology, Cytomechanics, Substrate strain, Cell response to strain

## Abstract

The author started a niche biotech company in 1985 called Flexcell^®^ to distribute an enabling technology, mechanobiology devices, to the field. He was the first University of North Carolina faculty member to start a company and stay with it as he pursued his career in academics. That was an unpopular route at that time, but a path he was driven to navigate. Those interests, merged with his training, led to the design and manufacture of mechanobiology devices such as the Flexercell^®^ Strain Unit and the BioFlex^®^ flexible bottom culture plates to study fundamental responses of cells to strain. Principles in these devices were also incorporated into bioreactors for tissue engineering, which are standard in the marketplace today. In this article, the major roadblocks will be chronicled that were overcome to help build the field of mechanobiology and create a small biotechnology company. Through example, the author’s formula for achieving milestones will be discussed including, the DRIVE it takes to get there [“DRIVE”: Determination (Confidence), Research and Development (R&D) and Risk-Taking, Innovation (Imagination) and Intellectual Property, achieving Victory, and Enterprise].

## Determination

The author grew up in McKeesport, PA right outside Pittsburgh, a hub of US industry, especially steel making in those days. Ingots of red hot steel would stand like fiery soldiers against the background of the blast furnaces, rails and freight cars in the switching yard just off Lyle Blvd. Sometimes, you would wait for 20 min while the freight cars rolled by with their cargoes of coke, ingots, and machined parts, moving all over the country. If you weren’t curious about what the cargo was on those cars, you did not have much of an imagination. On weekends and during summers off from Shady Side Academy in Fox Chapel, there was work in his uncle’s garage on cars, changing tires, fixing brakes, doing minor engine overhauls, and washing cars in the BKL Car Wash when it was all manual (Banes, Kohut, Lenhart). Later, on summer break from Lehigh University, work involved cutting right-of-ways, digging ditches for the Equitable Gas Company, and finally doing autopsy openings and histology with Dr. Cyril Wecht, as the first temporary laborer in the Allegheny County Coroner’s Office, PA. If there was any free time left over, wood was cut for the fireplace, doors were painted, and this or that were fixed. There was serious study at Lehigh but 4–6 h/day, were spent all year round, in the gym or on the track in the author’s event, the pole vault. It was all great preparation for what was to come.

In 1969, as a senior at Lehigh University in Bethlehem, PA, the author observed a graduate student engineer in Dr. Bradford B. Owen’s comparative anatomy lab in the biology building painting a cow hock bone with a green epoxy material in preparation for a compression test. He explained what should happen with strain marks in the epoxy and bone surface under compressive load. In graduate school, for his Master’s degree in developmental biology, author built micro-dissection tools for embryology to ablate chick or mouse embryo tissue. For his Ph.D. at the Medical College of Virginia microbiology, he built tube, then slab gel electrophoresis units and sold them to professors for their research. Pharmacia Inc. picked up on the colored plastic in the electrophoresis units to jazz up their commercial counterparts. As an assistant professor at UNC, he designed an auto-hydrolysis device to automatically acid or base hydrolyze and dry multiple protein samples for subsequent amino acid analysis. A company in New York took the design, patented the device and built the units without his consent. Later when they had a problem with function, they called for help. They were advised to drop them off the Tappan Zee Bridge! Varian Inc., chromatography division, later picked up their patent. In retrospect, there were not many fellow students or faculty members who did these things. So, as a 27 year-old post-doc, sitting in a UNC lecture hall thinking about how to stretch cells with some kind of device, he determined how to go about doing it, and then he did it.

The “modern” field of mechanobiology is now about 40 years old. *The bone people started it*. They will cite Julius Wolfe and the 1892 thesis of bone strain followed almost 100 years later by Harold Frost and the mechanostat theory.^8^,^13^,^27^ *The blood flow people started it.* 7,18 Early fluid dynamics researchers were in the literature investigating blood flow in tubes and later laminar shear stress. *The biomechanics people started it too*. Many engineers were interested in strains on tissues and materials.^6^,^9^,^12^,^28^ In fact, Bob Nerem held one of the early special interest group meetings on deformation and cell response at Georgia Tech (1991), preceded a few years by the National Heart and Lung group that held a meeting at the NIH on stretch effects and lung (NIH Lung Institute, 1989). As a post-doc at Duke University in the Microbiology and Immunology Department in 1976, the author attended a seminar at UNC by Itzhak Binderman DMD, an Orthodontist Clinician-Scientist from Tel Aviv University, on stretching osteoblasts by applying tension to the bottom of a culture plate with an orthodontic jack.^13^ The cells showed an increase in Ca^2+^, cAMP and PGE2 release. This research was really ground-breaking work! As a former pole vaulter, the author compared what a fiberglass pole would do under flex and knew that the polystyrene could not stretch much. The inner surface of the culture dish would likely be in compression not tension. Everything in the author’s life came together at that moment—all the years of athletic endeavor and love of science. He was writing, excitedly, in his idea book, that a far better method would be to grow cells on a flexible surface and stretch them by vacuum, from below. His theory was that cells should be exercised in culture, not grown on static substrates, and he wanted to elucidate the mechanisms by which cells responded to strain. That concept turned into a design, then and there, with prototyping a Plexiglas baseplate and rubber gasket in the shop at the Dental School at UNC. It took 3 years to reduce the idea to practice and until 1985 to commercialize it. Meanwhile, work continued on soon to be funded collagen work with bone and tendon.

## R&D: The Science and Engineering

The author was off on a commercialization path, alone. He worked very hard selecting a flexible material that could sustain cultured cells, from latex to urethane to silicone rubber. Lab gloves and any source from the McMaster Carr catalog or chemical companies were fair game. Hailing from PA, he thought it best to start in the more industrialized north, New Jersey, Ohio, PA where there were old plastic moulding operations and new engineering companies taking advantage of the upstart digital computer age. He had a fundamental design on graph paper for a multi-well, flexible bottom culture plate but no place to take it. Most universities were just starting their “biotechnology” programs after the models of MIT, Boston University, Stanford and UCLA. The University of Pittsburgh had the Pittsburgh Tissue Engineering Initiative (PTEI) started by Peter Johnson MD, a plastic surgeon who saw the light with tissue engineering (http://www.ptei.org/). The State of Pennsylvania had the Ben Franklin Technology Partners (benfranklin.org/what-is-bftp), which provided funding for Pennsylvania-based start-up companies (subsequently reporting a 3.5:1 return on investment since 1983). Entrepreneurs need start-up funding. The author had taken money out of his meager retirement fund (50% common stock) and Attorney Banes pitched in (50% common stock), but serious research dollars were needed to fund the development, characterization and validation of the cell strain device and the culture plate. A patent on the method for derivatization of silicone elastomer and the plastic culture plate design had already been submitted to the USPTO (United States Patent and Trademark Office). The Ben Franklin Partnership opened its doors for funding of start-up companies in Pennsylvania in 1982. Three applications were made and won by the author over the course of three years (1989–1991; $274,000 total) for characterization of the chemical surface of the culture plates and for a multiwell, flexible bottom culture plate to be used for drug studies. In 1990, an additional award was applied for and won through the North Carolina Biotechnology Center for equipment ($100,000 low interest loan paid back).

**Figure 1:**
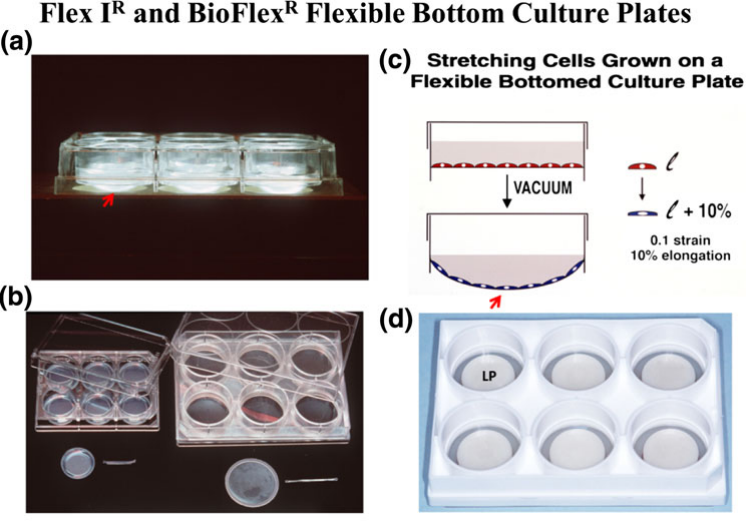
(a) A side view of the Flex I^®^, flexible bottom culture plate with cast, thick (0.020 in) silicone elastomer membrane seated on a natural rubber gasket. The red arrow shows the membrane deformed in unconstrained distension downward by the underlying vacuum. (b) A top view of the Flex I^®^ 25 mm diameter 6-well plate (left) and the BioFlex^®^ 35 mm diameter 6-well plate (right) with a top and side view of the silicone rubber membranes for each. (c) A side view diagram of a well with flexible substrate with cells in red at rest (top) and during active stretch (bottom). The red arrow shows the same location for the distended substrate as in panel a with *l*/*l* as strain. (d) The current embodiment of a BioFlex^®^ 6-well flexible bottom culture plate with a nylon LP beneath the flexible membrane. The perimeter of each well is open to vacuum that deforms the membrane across the LP face and yields radial strain across the substrate

The first flexible bottom culture plate Flexcell^®^ produced was a 6-well plate with a 25 mm diameter well (Fig. 1). The mould was produced in New Jersey under Bob Tilp’s direction at the machine shop of Springfield Tool and Die, Inc. in Union, NJ (Tilp Manufacturing Inc., the oldest plastic moulding plant in the east). A proper injection moulding tool, known as a “mud mould” with interchangeable inserts, was made so that solid bottom as well as flexible bottom culture plates could be “shot” in the same mould, the latter of which could capture a silicone rubber membrane that was molded as a cup-like construct. Plexiglas prototypes had already been designed and parts that screwed together, were made at a local machine shop. Testing several embodiments of rubber membrane capture mechanisms, showed that it was best to build “O” rings into the plastic rather than have “O” rings in the rubber “six pack”. At this point, a referral from the Ben Franklin people led to a Pittsburgh company that was probably one of the few existing silicone rubber manufacturing companies of medical grade finished products, having made poppet valves for heart lung machines. A mould was purchased for $5200 to cast “6 pack” silicone rubber membranes. However, tolerances could not be held across 6 inches in molded rubber as one can with plastic. The molded membranes would not register with the “O” ring capture rings in the plastic without having sagging membranes with incorrect tension (quantified with a proximity detector). That concept was a learning curve lesson. So while the world of silicone rubber sheeting matured, Flexcell moved forward with the original plan of casting membranes by mixing medical grade Dow MDX 442-10 and platinum catalyst curing agent in a material mixing machine (North Canton, OH), the same kind of machine used to mix epoxies to fill golf driver heads. More risk was taken and the mixing machine was purchased for $15,000 with air driven mixers and a degasser to remove air from the molasses-like rubber mixture. It worked! Previously, the materials were hand-mixed then centrifuged to eliminate entrained air. Next, a bottom was needed to support the rubber membrane as it cured. Scotch-type wide tape was used to cover the bottoms of the Tilp-moulded, Flex I^®^ open-bottom culture plate bodies, then 1 g of mixed, degassed rubber was dispensed per well. The sidewalls of the wells were coated in a reproducible way, by using the Flexercell^®^ Strain Unit program to run a motor in a timed fashion to rotate a tilted tray full of Flex I^®^ plates to run the rubber material up the sidewalls allowing a cup-like geometry and mechanical adhesion by dry tack to the plastic sidewall. In the end, a six-well rubber bottom culture plate with a “thick” membrane was born that would withstand thousands to millions of repeated downward flexes. This was the first production Flex I^®^ culture plate!

Getting cells to adhere to silicone rubber was no small feat. Dow chemical was called for advice, but the breast implant problem was in full swing, and no information was forthcoming. It was difficult just getting the reagents to make medical grade silicone rubber. Polystyrene culture plates were treated to make the surface hydrophilic, but none of the culture plate reps were talking about how that was done. A paper came to the author’s attention about a microbiologist using “glow discharge” to treat plastic to increase cell adherence. Therefore, companies were contacted involved in gas plasma technology. The Branson Inc. rep informed us about the equipment needed to decrease the hydrophobicity of the surface (decrease contact angle to about 80°). A cold gas plasma unit and high capacity vacuum pump were another $60 k (used equipment). Interestingly, when an oxygen gas plasma was first used to treat silicone rubber, it was found that just a few seconds of plasma exposure would make the surface hydrophilic and supported fantastic cell adhesion and spreading. Unfortunately, this reaction was transient as the groups were lost after about 72 h. Starting up the first oxygen plasma and the 100 ft^3^/min vacuum pump was exciting! Fomblin oil was used (explosion-proof and costly) in that pump to reduce the chance of explosion, but it was a bit tense when the system was started. We weren’t quite sure if the building or contents would survive! The next challenge was the silanol chemistry of the silicone elastomer membrane. The chemistry department at UNC was contacted but no one knew much about this chemistry at the time. The library (no internet then) was the next intellectual repository researched and a great book on silicone chemistry was found with 15,000 possible compounds that might be used to derivatize the base silanols. The compounds were scanned, 30 were selected and 8 were purchased and tested. Five compounds worked well, but triethoxysilylpropylamine (TESPA) worked best. Upon reading the material data safety sheets on the compound, it was found that TESPA was the compound of choice for derivatizing glass! That compound was used as a base reagent to obtain a derivatizable amino group which could then be used to react with other groups, especially the then new RGD group (arginine, glycine, aspartic acid) peptide that was just recognized as an integrin binding peptide needed for cell attachment to a surface.^20^ Two grams of RGD with various spacer groups were ordered from the then new peptide facility to present RGD at least 20 angstroms from the silanol surface. It worked! Collagen peptides were already used to covalently bond to the rubber surface, which worked well. The reaction to the initial observation of stretched osteoblasts in 1985 with Mike Buckley, DDS MS was memorable. A collaborator and good friend, Professor Allen Boyde, a world renowned electron microscopist, was there visiting Jerry Mechanic and the author in the Dental Research Center (DRC). He came over to our lab at the very moment when we pulled our first osteoblast cultures out of the incubator after a “night’s stretch”. Upon observing the cells, the author exclaimed, “Geeeez look at that!” Mike looked and said, “What?” The author replied, “Don’t you see it?” Allen gave a look and in a second, said, “They are all aligned in the perimeter!” Wide-eyed, Mike inspected again and exclaimed, “They are, they are aligned in the perimeter!” That result seems simple by today’s standards, but we were off and running. Mike continued to look at osteoblast response to strain.^4^,^5^ Linda Levin, DDS, Ph.D., then in the lab as a post-doc, worked on possible heat shock protein induction by strain in periodontal ligament fibroblasts^16^ (she won an NIH post-doctoral fellowship). Later, Bauer Sumpio, M.D., Ph.D., on a vascular fellowship with George Johnson, Vice Chair of the Surgery Department at UNC, worked on strain response to endothelial cells and smooth muscle cells.^22^–^25^ The author was intent on proving that there was a field theory that could explain a cell’s response to strain (*R* = summation of (*ε*, *f*, *t*) where *R* is the cell response, *ε* is the strain magnitude, *f* the frequency of the deformation and *t* the duration of the event).^2^

## Hardware and Programming

The author learned automation from his post-doctoral mentor, Jerry Mechanic, who built the first flow-through scintillation counter using a Beckman counter and stop-flow technique to quantitate collagen crosslinks in matrix.^17^ This was prior to the digital age, and strictly analog. Then came the Texas Instruments hand held computer (TI-59) with “chewing gum wrapper card” (named for the card’s size) for algebraic-like programming. One could easily make up an algorithm to do any simple or complex calculation up to 999 steps (whose limit was approached once!). Ollie Montbureau, the Dental Research Center shop electrical wizard, belted this device to our BD spectrophotometer to record absorbance on the printer tape without stopping. The boat was missed there as there were no such devices at the time!

**Figure 2:**
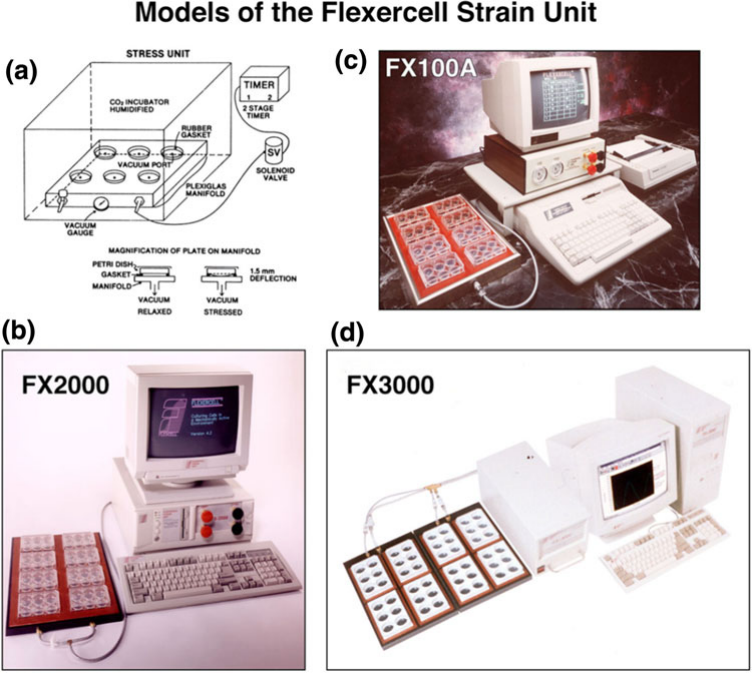
(a) A schematic from the first prototype strain unit that provided regulated pressure to the bottoms of gasketed culture plates in an incubator. The timer controlled the voltage to the nominally on/off solenoid valve which controlled the vacuum level to the baseplate on which a rubber gasket with culture plates resided. Vacuum deformed the plate bottoms and thus the substrate on which the cells were adherent and placed a proportional strain on the cells. (b) A Flexercell^®^ Strain Unit *ca.* 1994; (c) *ca.* 1985–1993; and (d) *ca.* 1995–2000. The single baseplate with 8 Flex I^®^ culture plates (25 mm diameter wells) was placed in a CO_2_ incubator for culture and had connecting tubing to the controller unit run out a bunged hole in the incubator top (b, c). (d) Two baseplates each with four BioFlex^®^ 6-well culture plates (35 mm diameter wells). The current strain unit is a model FX 5000 with touchscreen and addressable, proportional valves (not shown)

The first prototype cell-strain unit the author built had a complex timer and a single valve and manually operated bleed valves to control input and output air to the bottoms of plastic then rubber bottom culture dishes made in the shop (Fig. 2a; FX100A, Flexercell^®^ Strain Unit). Red Hat valves were selected and a simple minded piping system was devised to control air movement-vacuum to deflect the underside of the culture dish downward, and air release to complete the half-sinusoidal curve representing the strain event. Then came the first cost effective computers, the VIC 20, Commodore 64, then Radio Shack, IBM, DELL and others. Apple was not used due to expense. An EPROM was burned with our program that was the forerunner of the current valve control code. Later came C+, C++ and more complex codes. Finally, a commercial firm in Pennsylvania (Remote Control Inc., Ron Coiner, Irwin, PA, with two partners who broke off from Westinghouse Air Brake Co. and had a combined 60 years experience in pneumatics) took over the programming control and unit fabrication for a model FX-3000. They fabricated sub-assemblies which were then completed as FX3K units, calibrated and tested in NC. To gain better control of the assembly process, a firm in NC was contracted to provide sub-assemblies, then total assembly capability was brought in house later. As components changed, software patches were programmed to accommodate new parts. Eventually, the software was totally rewritten, and a most trusted friend and programmer par excellence helped us with this project. This was Elliott Green, Ph.D., a friend and colleague from graduate student days at the Medical College of Virginia. Elliot reviewed the current software, and in a marathon of coding, knocked out our new program (FX4K). He maintained that a more important contribution to the success of this next generation Flexercell Strain Unit was codifying the granularity in the hardware to understand the limits that one could achieve in the system calibration. The most important changes occurred with software updates to control the pressure transducers, the invention and use of addressable, proportional valves (vs. nominally open or closed valves) and the use of wireless technology and an internet connection. All these new hardware components required more than patches to the program and forced a more modular design to the software architecture to accommodate future changes.

## The Mechanobiology Field

**Figure 3:**
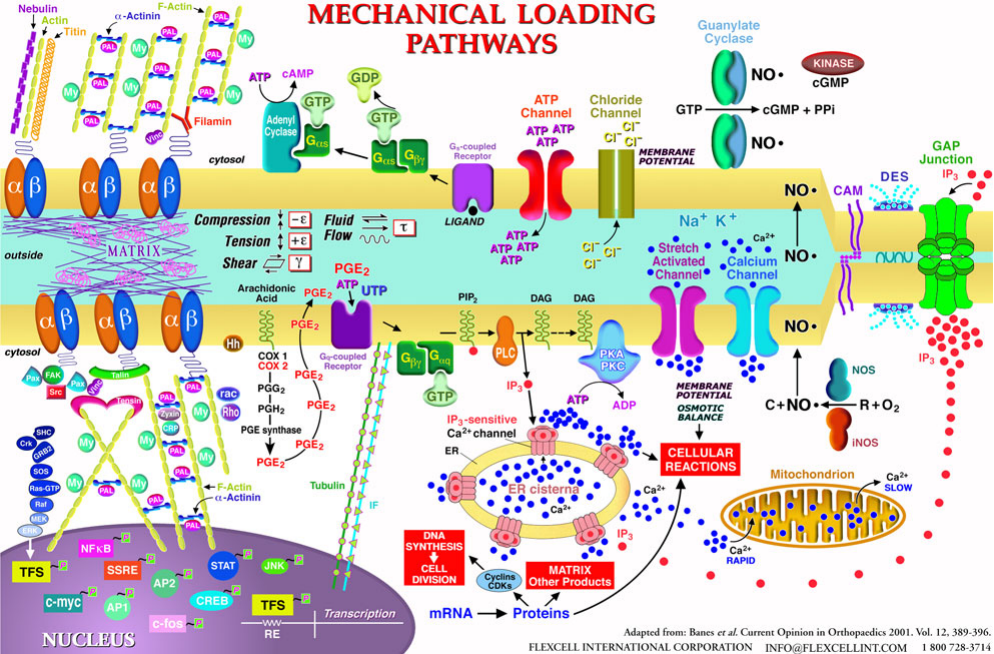
A view of the redundancy in how cells respond to mechanical loading (*ca.* 1995). Response pathways included (1) through the matrix to integrins to the cytoskeleton to the nucleus, (2) *via* stretch-activated channels, (3) purinoreceptors, (4) connexins, (5) CFTR channels, and (6) calcium channels, among others. A primary cilium (not shown) would be present today with an unresolved mechanism for where it fits in the hierarchy of responses

Mechanobiology was a difficult sell for many scientists when Flexcell opened its doors (*ca.* 1985). The author was an enthusiastic promoter of the cell stretching concept, the ideas that cells had redundant mechanisms to deal with strain and that cells should be cultured in a mechanically active environment as the standard. There were vigorous attacks for using hyper-stretch by some, especially in the osteoblast field. It had been shown that osteoblasts in bone routinely were exposed to 300–500 microstrain and strain at bone fracture was about 3000 microstrain.^21^ However, the argument was that these strains were measurements on bone, the material, not at the cellular level. A provisional matrix in most tissues has very high strain levels. Steve Goldstein of the University of Michigan at Ann Arbor substantiated this point in a rather heated debate at a meeting (“Bridging the Gap” co-host Professor Lutz Claes, head of the Biomechanics Institute in Ulm, DE) in Regensberg, Germany in 1993 when this argument came up again. It was Steve who made the fracture callus analogy bearing up to 50,000 microstrain, according to his measurements. He had designed and built a mechanical loading device that was implanted in bone and could be externally controlled to increase force in a living animal. He showed that one could apply controlled strains and get a biologic response *in vivo*. Moreover, responses to applied strain were too important to be relegated to a single pathway which substantiated the theory of redundancy in response pathways (Fig. 3). Don Ingber had an early theory concerning the cytoskeleton and the link to the cell as a tensegrity structure.^14^,^15^ There was agreement with this model but direct mechanical linkage could only take one so far. What about ligand mediated effects, ion channels and cell contraction (chemical-mechano response), differing parts of a cell with diverse intrinsic strains and now the role of the primary cilium? Responses to strain are indeed hierarchical and diverse.

## Characterizing the Strain

The next real challenge and controversy came with characterization of the strain in the Flex I^®^, thick rubber membrane (1.5 mm then vs. 500 microns today) and then in the cells themselves. The problem was approached from four directions. (1) The shop made a round, aluminum jig with concentric rings 1 mm apart, north, south, east, west axes and axes offset at 45° from the principles axes, to fit inside one well of the Flex I^®^ culture plate (25 mm diameter). Rubber membranes were molded in the Flex I^®^ body and the ring and axes jig was inserted onto the membrane before the rubber set, to imprint the impression of the pattern in the surface. Upon rubber setting, a curve was made of input vacuum vs. strain in the membrane by taking a dental material impression of the distended membrane (in unconstrained distension) to establish pressure vs. strain at points on the membrane. (2) A consult was held at Duke University with Bob Hochmuth, then chair of Mechanical Engineering, about the problem of mathematically solving for the strains in the membrane. He suggested Roger Tran Son Tay, a Vietnamese engineer in the department to address the problem. Roger was an amiable and willing collaborator and arrived at solutions for thick and thin plates mathematically.^26^ (3) Finite element analysis had just arrived in a software package from Swanson Analytical Systems, and Jerome Gilbert had just come from Duke Biomedical Engineering to Orthopaedics at UNC. The problem was addressed with the FEA package with the delightful finding that Roger’s solution was concordant with Jerry’s! (4) Lastly, strain gauges were bonded to the underside of polystyrene culture plates to repeat what Itzhak Binderman had done in 1976, and compression was measured in the plate center when an orthodontic jack was used to apply force to the plastic (bent like the pole vault pole facing the vaulter). However, it was confirmed that strains in the center of the rubber membrane were negative (compressive) vs. positive and gradient toward the periphery.^11^ This presented a problem as the biomedical engineering community wanted well defined strain, both in grant reviews and manuscripts. The question was addressed from the standpoint of how to image cells on the membranes. That alone was a problem given the thick membranes. It seemed difficult to even “see” the cells without using a dissecting microscope. High resolution, phase contrast pictures were needed to indicate what the cell substrate looked like, especially since, in 1985, the author believed that focal adhesions (UNC’s Keith Burridge) were involved in the cell’s contact with the rubber growth surface and in response to strain (which indeed they are). The author’s lab had already shown differences in actin and tubulin shifts in strained cells and cell alignment.^1^ Keith took a look with his DIC microscope but could not see anything. The membranes were too thick. A return to the Olympus phase contrast microscope and more focus on how cells could be visualized bore fruit. Two ideas developed: Theodore von Karman’s thought to go with the theory and there was no way a rubber membrane was going to defeat me (“When in doubt, I go with the theory.” Theodore von Karman Ph.D. First Science Medal winner, 1962 presented by JFK, Top ten minds of all time, Hungarian, from, “The Wind and Beyond” by Lee Edsun, 1967)! The refractive index difference between rubber and glass was preventing direct observation of the cells, so a culture lid tray to a 6-well plate was filled with water and the Flex I^®^ culture plate was placed in this “water bath”. The assembly was placed on the cell culture microscope stage with light source and objectives above. Voila! The cells could be seen, but not so clearly. Next, the objective lens was unscrewed at the nosepiece until the cells came into view. Some microscopes did not have enough depth of field to focus, so depth was achieved by screwing out the objective lens until in-plane focus was observed. An extension ring was fabricated at the shop (like close-up rings for single lens reflex cameras) for the objective lens so that it did not have to be screwed out to focus.

## Confounding Shear Stress and Collaboration

About this time, the question arose of how much fluid shear stress might accompany the flexing membrane. The author served on the NIH Musculoskeletal Study Section headed by Dwight Davies of Case-Western Reserve in Aerospace Engineering then, and later Steve Goldstein of the University of Michigan, who started the BME program there. Tom Brown, Director of Research in Orthopaedics at the University of Iowa also served on this SS. Tom is a highly respected engineer specializing in wear particles and hip implants. Tom always gave thoughtful and excellent reviews of principally engineering grants, particularly those of Van Mow (triphasic theory) and Woo (ligaments and robotic control of multiple degrees of freedom). At a lunch table during a review session, the discussion turned to the need for this problem of shear stress to be addressed by a bone fide engineer. Tom did his Ph.D. in fluid mechanics at the University of Pittsburgh, so there was a Pittsburgh connection as well as a genuine interest in fluid shear stress. Together, a collaboration was forged to write a three year proposal to investigate the fluid dynamics and shear stress caused by accelerating the medium overlying cells with a moving membrane.^3^ The Banes’ lab would look at the biologic readout in fast (Src expression and some other early response genes) and slower responses, cell division and matrix expression. We got a 0.2% rank on that application! There was a lot of interest in the field, and Tom was an excellent grant writer! Tom’s FEA modeling analyses (with collaboration from Doug Pederson in Tom’s lab) combined with our cell readouts indicated that shear stress was not a significant confounding problem.

**Figure 4:**
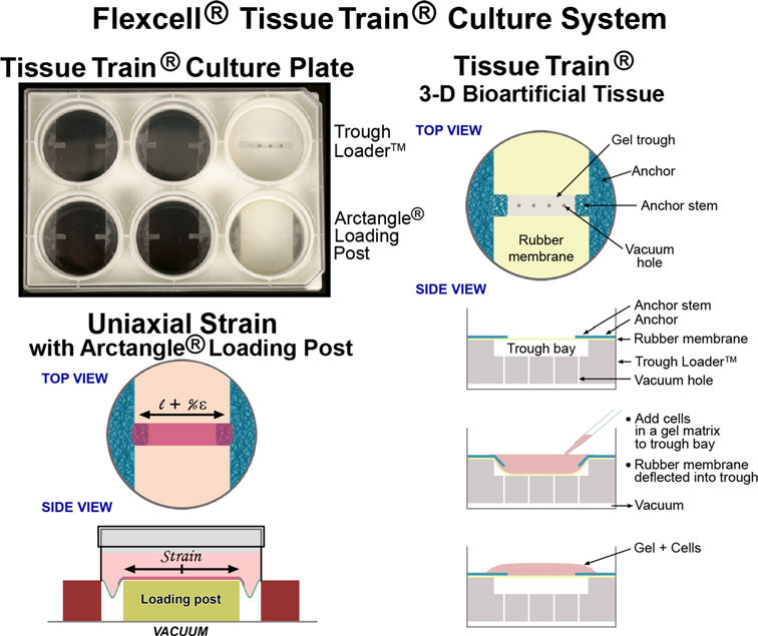
A top view down onto a Tissue Train^®^ culture well designed to fabricate a linear, three dimensional (3D) cell-populated hydrogel. The well is constructed with a silicone membrane sandwiched between polystyrene base and body culture plate parts. The blue mesh material signifies a nonwoven nylon that is bonded to the membrane only at the sector locations (anchor) at east and west poles. The rectangular anchor stems are collagen coated and free to engage a hydrogel, cast in the gel trough, in the central region between the blue tabs, created when vacuum deforms the membrane downward creating a void for casting using a “trough loader device” (top right of the six well plate on left). On side view, one can see the trough loader void into which the membrane (yellow) is deformed, creating the casting space for the linear, cell-populated hydrogel (pink, bottom right). Once the gel has set, the vacuum is released and the cell-gel construct rises to the membrane level. To apply uniaxial deformation to the bioartificial tissue (BAT), the trough loader is replaced with an Arctangle^®^ LP (rectangle with curved short ends, six well plate bottom right in plate) leaving the east and west poles beneath the nylon “open” to the underlying vacuum along the long ends of the LP. In this way, controlled, uniaxial strain can be applied to the BAT (bottom left, d*l*/*l*)

Based on analyses of the strain fields in the rubber membrane, it was understood that unconstrained distension of the membrane resulted in a gradient of strain in the membrane and to the cells. But this was only one way to strain cells. Investigators wanted to be more precise, with better defined, more homogeneous strain fields in the growth surface. This meant defined equibiaxial-radial-circumferential and uniaxial strain to simulate principle strains in organs like lung and the cardiovascular system and orthopaedics, respectively. The equibiaxial or radial strain requirement was the easiest to design. Planar faced cylinders were placed beneath each well of the BioFlex^®^ culture plate (Fig. 1d) and greased at the bearing surface with a silicone grease to allow gliding (Banes AJ. Loading station assembly and method for tissue engineering. United States Patent 6,472,202, Filed September 29, 2000, Issued October 29, 2002). Next came uniaxial strain and stretching cells in a 3D matrix that the soon-to-be burgeoning tissue engineering field would need. A circular, nonwoven, flexible but inelastic nylon mesh was bonded to the perimeter of the rubber membrane to capture a hydrogel in the nylon mesh. Vacuum deformation of the membrane was used to provide equibiaxial strain to 3D constructs such as dermal cultures. The cells occupied the central part of the gel and were strained equibiaxially as the nylon was deformed downward at the periphery over a circular loading post (LP)^10^ (Fig. 4). The uniaxial solution was a bit more difficult but interesting. A design called an Arctangle^®^ LP was used, comprising a rectangle with curved short ends that filled in the distance between the post and the BioFlex^®^ well perimeter at north and south poles, but left the east and west poles open to vacuum and thus, strain. A nylon mesh in the form of a sector with a tail, was bonded to the “open” east and west poles of the membrane so that vacuum could be applied to deform the membrane downward at the sectors. The tails at east and west poles distributed force to the linear construct, yielding uniaxial strain in the principle strain direction. There was a range of deformations, laterally, due to the opposing Poisson deformation, perpendicular to the principle strain direction.

So now there were 2D and 3D systems for applying cyclic strain to cells in culture. Disposable, dynamic culture plates and the strain machine were developed. Next came a device for applying compression (Compression +^R^) to tissues or cells in hydrogels, then a 6-chambered shear stress-providing device (Streamer^R^) for study of laminar, pulsating and reversal of fluid flow on cells and most recently, the HiQ Flowmate^®^, a syringe pump for microfluidics applications and more.

## Innovation and Intellectual Property

There was no help for an entrepreneur at our university in those days. There was a panel of 12 faculty members who reviewed the potential of 5 submitted proposals for inventions in 1985. The Information Technology Office and UNC’s Patent disclosure record and patents awarded numbers have improved since then with an explosion post-1985 (1960–1985: patents filed 39/issued 19 licensed 21; 1985–2001: filed 843/issued 298 licensed 553; see Table 1 for complete data to 2012). Two proposals from the Banes’ lab were submitted to the UNC IT office in 1985, one of which was the idea of the flexible bottom culture plate and a timer device controlling a pneumatic valve regulating pressure beneath the culture plate. Nine months into the university process, the author was anxious about a decision from the panel. The first paper on the stretching device and the idea of the flexible bottom culture plate had been submitted to the *Journal of Cell Science*.^1^ One of the reviewers commented that this novel method was going to be an important advance in cell culture and biology. The UNC IT office was queried on the subject of the IP, and the committee’s decision was not to pursue the topic at the university level. The patents were filed by Banes and eventually issued in 1988 forward, having taken a few years to process (Banes AJ. Biocompatible polyorganosiloxane composition for cell culture apparatus. United States Patent 4,789,601, Filed May 4, 1987, Issued December 6, 1988.) There were problems with intellectual property piracy that first year by a group who got two of the author’s grant applications on stretching cells and submitted their own application that year. The NIH proposal and submission were discovered after queries from a medical student from another institution who had worked in the Banes’ lab and who had discussed unpublished work with his mentors there. Banes spent $11 k on attorneys against the opposing university’s intellectual property attorney. It was a draw, but the interlopers withdrew their application from the NIH.

**Table 1:** Summary of activity for the University of North Carolina at Chapel Hill Office of Technology Development (by fiscal year)

	2000	2001	2002	2003	2004	20005	2006	2007	2008	2009	2010	2011	2012
Report of Inventions Received	113	115	119	86	124	113	97	113	122	137	125	142	161
Inventions Licensed	57	56	92	62	38	47	59	89	60	83	42*	45*	62*
U.S. Patent Applications Filed	110	115	107	86	105	119	113	109	121	107	125	124	124
U.S. Patents Issued	57	52	37	34	35	29	32	32	21*	19	28	33	32*
Start-up Companies	5	11	3	3	3	1	6	1	7	1	5	7	7
License Income	$952,585	$1,214,980	$1,284,058	$3,863,229	$3,943,996	$1,987,551	$2,248,240	$1,770,993	$2,779,463	$3,043,947	$2,600,000	$2,200,000	$2,466,866.32

*(2008 Patents include 3 service marks/trademark registrations); 2010–2012 subject to change, courtesy Office of Technology Development UNC, Peter Liao

## Victory: Taking it into a Business Incubator

There were competitors who emerged within a year of Flexcell’s roll out of its strain unit. The author was assailed with comments such as, “We’ll see who is around next year!”, reminding him of Nikita Khrushchev’s statement in 1956 to western ambassadors, “We will bury you!” The author took these comments personally and drove him to dig in and prevail. LiveCo, Inc. from Vermont had its one-off stretch device (Vitrodyne) which was effectively a linear tensile testing device with a piece of silicone elastomer on which to grow cells. Vitrodyne from LiveCo, Fritz Garrison (1988), Cell Kinetics from Herman VandenBerg (1988), Mike Bushman, John Frangos, Al Grodzinsky and others introduced cell stretching, shear or compression devices. Bose and MTS were late but serious entries.

Flexcell leased 2,000 sq ft in the newly renovated Cohn textile plant in Hillsborough, NC after requesting electrical, plumbing and HVAC upgrades on the space. As other companies dropped out or moved on, additional space was leased (current 13,000 sq ft). Accountants, banks, insurance companies, vendors have all been utilized and helpful through the years. Of a current 15 employees, several have been with us for over 27 years, but hirings and firings have been dealt with. Only a short drive from Durham or Chapel Hill, and with a wealth of highly trained technical people at hand, there was difficulty getting talent to journey even 15 miles from Chapel Hill to Hillsborough, NC!

## Enterprise, Inventiveness and Jobs

Vannevar Bush was the founder of Raytheon Inc., developer of a computing architecture that predated the web, head of all R&D during WWII, including the Manhatten Project and architect of the NSF and NIH (http://en.wikipedia.org/wiki/Vanneva_Bush). His opinions concerning education and practicality are poignant today in his report to the president on “a program for post-war scientific research” in the US. “The Government should provide a reasonable number of undergraduate scholarships and graduate fellowships in order to develop scientific talent in American youth. The plans should be designed to attract into science only that proportion of youthful talent appropriate to the needs of science in relation to the other needs of the nation for high abilities.”

Vannevar Bush, Science, the Endless Frontier, July, 1945. Professor Bush included this prefacing quotation from President Roosevelt from 1944, “New frontiers of the mind are before us, and if they are pioneered with the same *vision*, *boldness* and *drive* with which we have waged this war, we can create a fuller and *more fruitful employment* and a fuller and *more fruitful life*.” These lessons are valid now.

Al Mann and his group descended upon UNC about 10 years ago in an attempt to cut a deal to cherry pick commercially valuable IP in exchange for $100 M or so in funding (www.mannfbe.org). One comment by Mr. Mann in his address to the faculty was that a university was a poor place to commercialize anything. The author agreed. Then and now, many academics and universities are caught up in self-interest, let alone conflict-of-interest, to see a way to use innovation in their setting to act as an economic driver for their region. Once it was shown in the 1980s, that in Massachusetts, 1/3 of the state’s GDP was linked to spin-off companies, others scrambled to emulate MIT, Boston University, Stanford and a few others who had embraced innovation and entrepreneurship by capitalizing on discovery and spinning it out of the university setting to create value, companies and jobs (now quantitated).^19^ The Biotechnology boom was on! A case in point is the matter of progress in a university or government setting vs. an industrial setting with the pace of sequencing the human genome. The NIH effort and the Francis Collin’s group had a decade-long jump on Cetus and Craig Ventor in sequencing the genome. But Ventor’s shotgun DNA fragmenting and computer assisted, puzzle-piecing method revolutionized and accelerated the pace of sequencing and reduced the sequencing error rate. Ventor’s shotgun method was superior to the conventional method but was initially suppressed because it would put other labs vested in the old technique out of business (quote from Ventor’s book). After the dust settled, both men’s books underscored their different philosophies in life and science: Collin’s book, “The Language of God” is personal, philosophical and poignant and Ventor’s book, “A Life Decoded”, is personal, pointed and pugilistic. I recommend both to my BME students with Ventor’s providing an underdog point of view and a model for risk-taking. Both groups got to the same place and concluded successfully, but Ventor got there quicker due to his willingness to take on risk and entrepreneurial fire in the belly. The missing element in moving forward to start a company is a willingness to take on RISK. Most university academics are unwilling to do it, although they risk quality of life routinely in the pursuit of funding. You must be willing to try the 1½ gainer off the high board to start a business! At a recent meeting at UNC with our tech transfer agent, the author spoke with a colleague who had a neat culture plate invention. He wanted an outside investment in his device. My response was that he could get a part prototyped for under $1 k and that 10 parts should be tested, *etc.* He was unwilling to invest anything in his own idea. So as he was going out the door I jokingly said, “About the only way you are going to develop fire-in-the-belly for anything is to have a lunch at your favorite Mexican restaurant!” What is missing from the academic’s quiver is risk taking, “skin-in-the-game” and a willingness to invest in one’s own technology (a missing component from most university tech transfer deals with inventors).

This brings us to the Strumsky Patent Database and the Brookings Institute report, linking number of patents allowed for a geographic region such as Durham and Chapel Hill or Raleigh and Cary, NC and the economic impact on the local economy (Strumsky, Brookings Institute Report Feb 2013). The data indicate that a greater number of patents arise from highly populated metropolitan areas, especially when they are associated with universities that have top flight science departments. This report indicates that a successful start-up company needs three things: (1) a highly skilled workforce to fill positions, (2) proximity to research universities, and (3) funding sources. It also indicates a greater number of patents per capita occur in top 20, highly populated metropolitan areas such as #1, San Jose-Sunnyvale-Santa Clara, CA at 5,066 patents per million residents (data from 2007 to 2011) or #20, Minneapolis-St. Paul-Bloomington, MN WI at 945 (Brookings Institute Report, Strumsky patent database). The most telling data lie in Table 2, showing productivity growth in the top 20 metropolitan areas with patents per worker from 1980 to 2010 (Table 2, Brookings Institute Based on Strumsky Report).

**Table 2:** Growth in productivity in the 20 metropolitan areas with the largest increase in patents per worker (1980–2010)

	Change in patents per million workers, 1980–2010	Annual productivity growth, 1980–2010 (%)	Predicted productivity growth, 1980–2010 (%)	Change in bachelors degree attainment 1980–2010 (%)
San Jose-Sunnyvale-Santa Clara, CA	13,206	3.3	2.2	18.4
Burlington-South Burlington, VT	8,355	2.1	1.7	16.6
Corvallis, OR	6,644	2.6	1.1	11.3
Winchester, VA-WV	6,633	1.6	1.6	10.5
Rochester, MN	6,536	1.6	0.9	14.0
Charlottesville, VA	4,491	1.4	1.4	15.1
Poughkeepsie-Newburgh-Middletown, NY	4,219	1.8	1.4	12.7
San Francisco-Oakland-Fremont, CA	4,059	1.9	1.2	17.5
Blacksburg-Christiansburg-Radford, VA	3,709	1.3	1.2	11.5
Austin-Round Rock-San Marcos, TX	3,591	1.9	1.3	12.8
Santa Cruz-Watsonville, CA	3,547	1.7	1.1	13.7
Boulder, CO	3,182	2.3	1.8	20.6
Seattle-Tacoma-Bellevue, WA	2,957	1.3	1.5	14.8
Raleigh-Cary, NC	2,848	2.3	1.9	19.8
Ann Arbor, MI	2,602	1.1	1.5	14.7
San Diego-Carlsbad-San Marcos, CA	2,357	2.2	1.3	13.1
Durham-Chapel Hill, NC	2,212	1.9	1.5	17.8
Provo-Orem, UT	2,062	0.5	1.3	12.0
Portland-Vancouver-Hillsboro, OR-WA	2,056	2.5	1.3	13.9
Racine, WI	2,046	1.0	1.8	9.0
Average for top 20 metros	4,366	1.8	1.5	14.5
Average of all metro areas	395	1.4	1.4	9.7

Annual growth was largest in San Jose-Sunnyvale-Santa Clara with a 3.3% growth and 18.4% increase in BA degrees awarded

*Source*: Brookings analysis of Strumsky database, U.S. Census Bureau, and Moody’s Analytics. Patent totals for 1980 and 2010 are based on 5 years moving averages that end in those years, since patent data fluctuates from year to year. Figures are based on application year of patents already granted. Predicated industry productivity multiplies metro area employment shares by sector by national productivity for each sector. The growth rate is calculated using 1980 and 2010 measures

If you reside in one of these metropolitan areas of the country, you are seven times more likely to obtain a patent than otherwise. Moreover, if your company is successful in winning a competitive NIH SBIR award, your economic region will prosper fourfold from the government investment. The report concludes, that to maintain leadership in inventiveness and drive economic growth, there should be: (1) investment in the US research enterprise, (2) supply of STEM, skilled workers, in the science, technology, engineering and computer science fields, and (3) safeguards and high integrity for the patent system and intellectual property (Brookings Institute Report 2013). A recent executive summary from an NIH-sponsored workshop on the biomedical workforce indicates that graduating Ph.Ds in BME ($68 k) earn starting salaries that are less than those out of chemistry ($69 k), clinical health fields ($79 k) and far less than economics Ph.Ds ($100 k) (http://acd.od.nih.gov/Biomedical_research_wgreport.pdf). Moreover, about 26% of BME Ph.Ds of average age 37 years, are entering tenured positions in academics compared to 34% in 1993. The rub is that academic training for BME students reflects largely a career path for academics rather than the larger market in industry. This academic preparation trend should be modified with much more emphasis on concrete skill set mastery geared to the job market and accountability measures for educators (employment by graduates).

The author did not invent the kidney dialysis machine or a new vaccine. These would have been more admirable efforts than developing the first commercial cell stretching device. However, the struggle to move mechanobiology devices forward in the marketplace was, at times, even more difficult than developing projects of greater merit. I let nothing get in the way of my progress. I just did not quit! Pittsburgh’s industrial history, weather, my immigrant, never-say-die heritage and athletics taught me that.

So what is holding the USA back from future success? The US ranks lower than one might expect given our apparent focus on education in K-12. Our universities are ranked highly among the world’s best, but our students have fallen in scores and rank only 24th in science and math. We are lower in publications and patents per capita with Finland ranking first! Therefore, if prosperity equates with education, entrepreneurship and jobs, we are on the north side of a black diamond slope on one ski! We must correct this trend and as a Nation, invest more in job-related skill sets and basic AND applied research now to assure jobs today and tomorrow (fund at least to the 25% of grants submitted!).

Bill Gates and his foundation feel the same way (2013 annual report). They are working at the root cause of our education challenge to distinguish what separates an excellent teacher from a poor one. In the end, we must engage, mentor and build confidence in our students to face the challenges of tomorrow. Perhaps we should focus less on grades and more on skill sets and actual job performance in secondary and college education. *Functional learning and accountability* in a task must be placed high on our goal list. It will mean a total revamping of Arts and Sciences, but it must be done. More hands-on labs and internships are vital to build confidence and skill sets in our engineers.

As for entrepreneurship, you can teach didactics and even networking, but risk-taking is another matter. Perhaps leading by example is best here. Leadership is not for everyone, but we also need followers to complete the circle. Lastly, you do not need to be a 4.0 student to be a success. If you want to be an Entrepreneur, you must have “DRIVE”: Determination (confidence), Research and Development Skills (R&D) and Risk-Taking, Innovation (imagination), a desire for Victory, and build Enterprise! As educators, we need to lead, instill and develop DRIVE qualities in our students.
